# Lignin Derivatives in Beech, Eucalyptus and Oak Wood Hydrochar: Mild Solvolysis and Characterization

**DOI:** 10.3390/polym18202530

**Published:** 2026-10-15

**Authors:** Valeria Pierpaoli, Miha Grilc, Giorgio Tofani, Blaž Likozar, Edita Jasiukaitytė-Grojzdek, Manuela Romagnoli

**Affiliations:** 1 Department of Innovation in Biological, Agro-Food and Forest Systems (DIBAF), Tuscia University, Via San Camillo de Lellis, snc, 01100 Viterbo, Italy; valeria.pierpaoli@unitus.it; 2 Department of Catalysis and Chemical Reaction Engineering, National Institute of Chemistry, Hajdrihova 19, 1000 Ljubljana, Sloveniaedita.jasiukaityte@ki.si (E.J.-G.); 3 Center of Excellence for Low Carbon Technology, Hajdrihova 19, 1000 Ljubljana, Slovenia

**Keywords:** hydrothermal carbonization, lignin derivatives, eucalyptus wood, beech wood, oak wood

## Abstract

This study investigates lignin-derived fractions obtained from the hydrochars of beech (*Fagus sylvatica* L.), eucalyptus (*Eucalyptus camaldulensis*) and oak (*Quercus cerris* L.) following hydrothermal carbonization and mild acidolysis. The aim was to understand the structural, molecular, and thermal properties of the recovered material and to compare how botanical origin influences it. FTIR analysis revealed consistent aromatic signatures across all samples; HP-SEC revealed species-dependent differences in molecular weight distributions, although the overall molecular weight range remained comparable among species; 2D NMR further verified the lignin origin of the isolated fractions, while TGA indicated comparable thermal behaviour among the three biomasses. The whole study shows how hydrothermal carbonization followed by mild solvolysis produces lignin-derivative fractions, highlighting both the potential of this approach for lignin recovery and the need for more efficient methods to obtain higher-integrity lignin suitable for advanced material applications.

## 1. Introduction

Significant quantities of lignocellulosic residues are generated during wood processing [1], while substantial amounts of waste wood also originate from raw materials deemed unsuitable for higher-value timber applications due to their low quality [2]. Such residues are addressed primarily for the most to bioenergy production. However, substantial research efforts have been devoted to developing alternative valorization strategies capable of generating products with higher added value than bioenergy alone. Among these, hydrothermal carbonization (HTC) has emerged as a particularly promising technology [3], producing both hydrolysates, which can be further converted into high-value-added compounds [4], and hydrochar [5,6]. Hydrochar offers several advantages over biochar, particularly because it can be produced from wet biomass [7]. As a result, no energy-intensive drying step is required during feedstock pretreatment. Furthermore, the range of hydrochar applications is rapidly expanding. For example, hydrochar can be used as a solid biofuel [7], as a soil amendment in horticultural and garden applications [8], and as a component in innovative composite materials, such as formaldehyde-free plywood adhesives [9]. The structure and quality of hydrochar are highly variable and depend on both the nature of the original feedstock and the hydrothermal carbonization conditions employed [10]. Its chemical composition is of paramount importance, as it strongly influences the physicochemical properties and performance of hydrochar, thereby affecting its suitability for a wide range of end-use applications. Furthermore, the chemical composition plays a key role in determining the surface functionality and potential for surface modification, which are critical factors in many advanced applications. A deeper understanding of the chemical characteristics of hydrochar is essential to support the promising use of biochar and its applications and technological development in the coming years. In the case of lignocellulosic feedstocks derived from forest trees, it is evident that the hydrothermal treatment does not completely remove lignin from the biomass matrix. Indeed, the resulting hydrochar retains a significant proportion of lignin [11], suggesting that hydrochar itself could be considered an additional source of lignin for subsequent valorisation processes [11]. Moreover, tailoring the lignin composition and structural variability within hydrochar may provide opportunities to tailor surface functionalization strategies and enhance the material’s performance in targeted applications. Lignin in hydrochar could be an interesting building block; it is the most abundant aromatic biopolymer in nature–its availability exceeds 300 billion tons, increasing annually by around 20 billion tons [12]. Structurally, it is a heterogeneous polyphenolic network mainly built from three monolignols-*p*-hydroxyphenyl (H), guaiacyl (G), and syringyl (S)-linked predominantly through β-O-4 bonds, among other interunit connections [13,14,15,16,17,18]. The relative abundance of these monomers and the distribution of linkage types differ across biomass sources (softwoods, hardwoods, grasses), resulting in notable variations in lignin’s chemical reactivity and mechanical behaviour [19,20]. Its functional groups–methoxy, carbonyl, carboxyl, and hydroxyl moieties–provide key reactive sites for chemical modification, enabling processes such as functionalization, depolymerization, and controlled fragmentation [12,20,21,22]. Modified lignins have been successfully applied in phenolic resins, adhesives, bioplastics and even in the development of nanostructured materials for drug delivery [23,24,25,26,27,28,29,30,31,32]. The reactivity and processability of a lignin depend on its botanical origin and on the severity of the treatment used to isolate it, which is why recovered lignin fractions are usually characterized in terms of molecular weight, phenolic hydroxyl content and thermal behavior before use [25,31]. This is particularly relevant because lignin is generated in very large amounts by pulping and biorefining but is still mostly burned as a low-value fuel [23,29]. Forest biorefineries therefore aim to convert lignocellulosic wastes, lignin included, into bioproducts and biomaterials [30], while lignin recovered from agricultural residues or green-solvent pretreatments can be valorized into higher-value materials, including bioplastics [27]. Bio-based wood adhesives are among the most relevant outlets. Phenol-formaldehyde resins rely on fossil-derived phenol and can release formaldehyde, whereas the phenolic structure of lignin makes it a candidate for partial phenol replacement [23,32]. Isocyanate-based polyurethane adhesives raise comparable health concerns: because industrial lignin is condensed and poorly reactive, chemical modification is usually required to make it suitable for these uses [28,32]. For example, in-situ phenolation of lignin during organosolv fractionation of spruce increased its phenolic hydroxyl content and led to an aldehyde-free plywood adhesive meeting the requirements of the relevant national standard [32]. Similarly, electrochemical oxidation of organosolv beech lignin demethylated and hydroxylated it, and the modified lignin was successfully used in non-isocyanate polyurethane adhesives for particleboard [28]. Modification also tailors lignin for other classes of materials. Esterification of kraft lignin with cholesterol gave it a defined melting behavior, which is relevant for melt blending with non-polar synthetic polymers, and the modified lignin self-assembled into nanoparticles able to load and release a hydrophobic model drug [25]. More broadly, the antioxidant and antimicrobial properties, biocompatibility and ease of functionalization of lignin support its use in drug and gene delivery and in tissue engineering [23]. For instance, lignin nanoparticles incorporated into electrospun polycaprolactone fibres imparted antibacterial and antioxidant activity and promoted mineralization and osteogenic differentiation, although with some loss of mechanical performance [24]. Finally, lignin-derived carbons with tailored porosity are used as electrodes in supercapacitors and in Li-, Na- and K-ion batteries [29].

Based on these premises, the present study aims to further investigate the characteristics and potential applications of lignin extracted from hydrochar derived from forest biomass. As a first case study, European beech (*Fagus sylvatica* L., hardwood) was selected for investigation. European beech is one of the most important forest tree species in Europe owing to its wide geographical distribution and considerable ecological and economic significance. It occupies more than 11 million hectares across the continent [33] and represents a key component of many forest ecosystems. In the Mediterranean region, particularly in the Italian Apennines, beech forests have historically supported the production of furniture, charcoal, firewood, and sawn timber. In recent years, increasing attention has been devoted to the valorisation of beech wood beyond its traditional use. Owing to its favourable mechanical properties, beech has been recognized as a promising material for structural applications and engineered wood products, including cross-laminated timber (CLT) and other advanced wood composites [34]. Consequently, novel valorisation pathways are currently being explored to promote higher-value uses of beech wood and reduce its reliance on conventional energy-related applications, such as firewood production.

Another group of species of considerable global importance belongs to the genus *Eucalyptus*, which is widely utilized, particularly for pulp and paper production. Eucalyptus has been extensively cultivated in Italy and is present in numerous regions across the country [35]. There is an increasing need to review the role of this species and, where necessary, replace it with more suitable alternatives, while exploiting the use of its wood; therefore, wood of low quality and residues from wood processing can be properly evaluated.

Finally, Turkey oak wood (*Quercus cerris* L., hardwood) is considered because forests occupy more than 900,000 ha, which are managed either as coppice or high forest stands. However, the prevalence of coppice management results in the wood being used mainly for fuelwood production, although this resource could be exploited more effectively in higher-value applications. Despite its abundance, it remains underutilized, and studies on its wood chemistry are limited, highlighting the potential for valorizing this local resource through innovative applications.

Despite the growing interest in HTC and lignin valorization, most studies have focused either on the production and applications of hydrochar or on lignin isolated directly from raw biomass. Comparatively, less attention has been devoted to lignin-derived fractions recovered from hydrochars, particularly when different hardwood species are examined under comparable processing conditions. Furthermore, the extent to which the botanical origin of the biomass influences the structural, molecular, and thermal characteristics of these recovered fractions remains insufficiently understood. Addressing this knowledge gap is important both for understanding the fate of lignin during HTC and for assessing the suitability of hydrochar-derived lignin fractions for future valorization pathways and advanced material applications.

Therefore, the aim of the present study was to characterize the wood materials of beech, eucalyptus, and oak lignin derivatives right after hydrothermal carbonization and solvolysis, similarly to what was already done with chestnut wood [11,36]. The research was intended to expand the research on three feedstocks very widespread in Europe and worldwide, with the aim of comparing how hydrothermal carbonization affects their composition and properties. Such a comparative approach will allow us to assess whether the resulting biomasses can be used for different or similar purposes and how specific functionalization may expand their potential applications.

The methodology employed will enable a comparison with the study conducted on chestnut [11].

## 2. Materials and Methods

### 2.1. Materials

*Eucalyptus camaldulensis* (Dehnh.) trees were sampled from the windbreaks of Tarquinia (VT, Italy). The mean diameter of the trees was 36.8 ± 10.3 cm and the mean height was approximately 20 m. From each tree, one log approximately 50 cm in length was cut. Boards about 4 cm thick were then prepared and subsequently seasoned.

The studied oak wood was derived from a disk of an adult tree of *Quercus cerris* L. from the Roccarespampani Estate close to Tuscania (VT, Italy). Flat-grained beech (*Fagus sylvatica* L.) wood samples, 15 mm × 15 mm × 3 mm (L X T X R), were prepared along the tangential direction from one cut board. All the wood materials were air-dried for several weeks and cut to pass through a 35-mesh sieve in an IKA M10.1 cutting mill (IKA^®^-Werke GmbH & Co. KG, Staufen, Germany), resulting in a wood powder with a particle size of approximately 0.25 mm that was put into the 250 mL hydrothermal reactor at 200 °C.

### 2.2. Hydrothermal Reactor

The hydrochar was obtained as a solid product of a hydrolysis conversion carried out in a Parr 4560 mini-batch reactor following Mohan et al. methodology [37]. The reactor was equipped with a glass insert with a solids loading of 10 wt% (oven-dried basis) in a 5% (*v*/*v*) sulfuric acid solution. H_2_SO_4_ 99% used was from Sigma-Aldrich, Milan, Italy. Prior to heating, the reactor was purged with nitrogen gas while stirring at 200 rpm for 15 min, followed by pressurizing the system to 300 psi and then releasing the pressure to ensure oxygen was removed from the system. The reactor was heated at a linear rate of 2.3 °C/min and held at 200 °C for 90 min and then passively cooled to room temperature prior to opening the reactor. Once the reactor was cooled to 50 °C, the solid-liquid slurry was separated using vacuum filtration through a 0.22 μM PVDF membrane filter (Sigma-Aldrich, Milan, Italy). The residual biomass cake was washed with deionized water.

### 2.3. Mild Acidolysis

The hydrochars were cooked under reflux using a dioxane (Sigma-Aldrich, Milan, Italy) -water (400 mL) in a volumetric ratio of 82:18 in a 0.1 M HCl solution for two hours. At the end, the solution was filtered for removal of the acidolysis residue (namely, "exhausted"). The filtration residue was washed with a portion of 200 mL dioxane-water (82:18 vol%). In order to precipitate the dissolved lignin in the filtrate, dioxane was removed by rotary evaporation and replaced with water of equal volume fractions. Finally, the solution was vacuum-filtered, and the collected lignin was further washed with acidified (pH 2) distilled water to remove sugar residues. Lignin was then put under the fume hood for 2 days and left to dry.

For each wood species, hydrothermal carbonization and the subsequent mild acidolysis were performed on a single independent preparation. Likewise, FTIR, HP-SEC, HSQC NMR, and TGA analyses were conducted as single measurements. Therefore, the results should be regarded as exploratory, and comparisons among species are qualitative rather than statistically validated.

### 2.4. Fourier Transform Infrared Spectroscopy (FTIR)

Samples of the hydrochar powder after the hydrothermal treatment and after the mild acidolysis for both the hydrochar and the extracted lignin were analyzed through FTIR.

Fourier-transform infrared (FTIR) spectroscopy was acquired using an FTIR ATR Bruker Alpha P spectrometer (Ettlingen, Germany). ATR spectra were collected with 4 cm^−1^ resolution, and the results were averaged over 64 scans with manual baseline correction and normalization.

Although the infrared scanning was done in the region between 4000 and 400 cm^−1^, data evaluation was limited to the fingerprint region (1800–900 cm^−1^) in which most of the variations of infrared absorption occur.

### 2.5. Solubility Tests

5 mg of each sample was weighed and placed in 1 mL of DMSO 99.9% (Sigma-Aldrich, Milan, Italy) in separate glass vials. The mixtures were left at room temperature for 24 h to ensure optimal interaction between the solvent and the sample. Then, the solutions were visually inspected for any solid residue.

### 2.6. High-Performance Size Exclusion Chromatography (HP-SEC)

The molecular weights of the lignin derivatives in the hydrochar after the mild acidolysis were determined by HP-SEC. A size-exclusion chromatographic system (Vanquish, Thermo Fisher Scientific, Waltham, MA, USA) with a UV detector set at 280 nm was used to conduct SEC on a PLgel 5 μm MIXED-D 7.5 × 300 mm. Polystyrene standards with molecular weights ranging from 672 Da to 127 kDa were used for calibration. Furthermore, guaiacyl glycerol-β-guaiacyl ether, which has a molecular weight of 320 Da, was used as a low molecular weight reference. Lastly, PSS WinGPC Unity software (Version: 1.0; Build 10478) was used to process the chromatographic data. The concentration of the samples was 1 mg/mL in THF 99.9% inhibitor-free (Sigma-Aldrich, Milan, Italy), and for PS standards, 1.5 mg/mL in THF 99.9% inhibitor-free (Sigma-Aldrich, Milan, Italy). Because the calibration was performed using polystyrene standards, the molecular weight values reported for the lignin derivative fractions should be regarded as relative molecular weights rather than absolute values.

### 2.7. 2D HSQC NMR

2D heteronuclear single quantum coherence (HSQC) NMR spectra of the lignin_char samples were recorded on a Bruker AVANCE NEO 600 MHz spectrometer equipped with a BBFO probe following the reported protocol [38]. Approximately 80 mg of the sample was dissolved in 0.60 mL of DMSO-d6, which was used as an internal reference point (δ_C_ 39.5, δ_H_ 2.50 ppm).

### 2.8. Thermogravimetric Analysis (TGA)

A Thermogravimetric Analyzer TGA/DSC 3+ STARe System (Mettler Toledo, Columbus, OH, USA) was used to investigate the combustion properties. The samples were placed in a 150 μL Al_2_O_3_ pan and heated from 25 °C to 800 °C at a heating rate of 40 °C/min under a nitrogen atmosphere. The samples were then cooled to 400 °C while maintaining the nitrogen atmosphere. Subsequently, the atmosphere was switched to oxygen, and the samples were reheated at 800 °C. A gas flow rate of 50 mL/min was used throughout the experiment.

## 3. Results and Discussion

### 3.1. Fourier Transformed Infrared Spectroscopy (FTIR)

**Figure 1:**
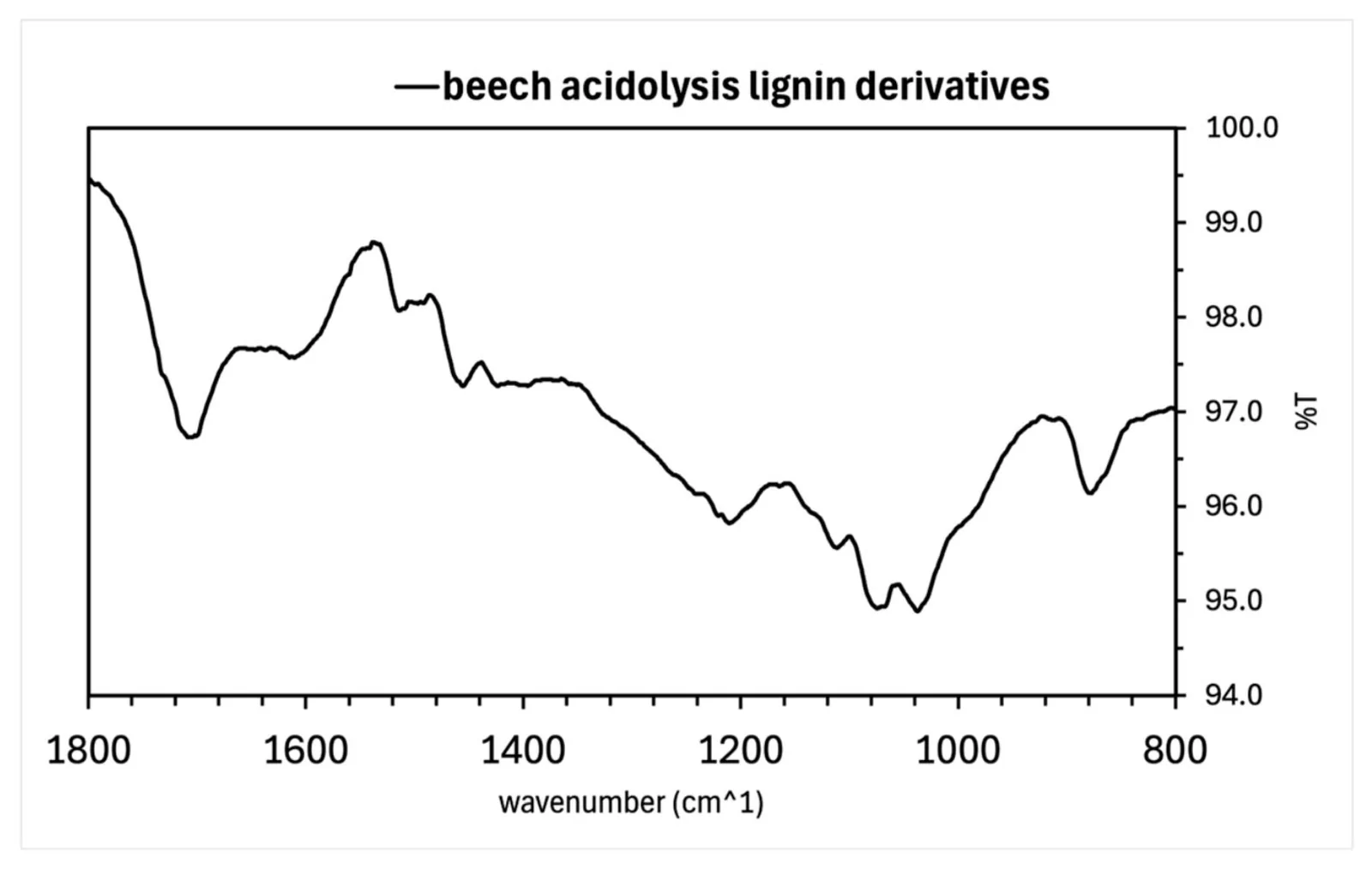
FTIR of the lignin derivatives fraction obtained after the mild acidolysis treatment of beech wood.

**Figure 2:**
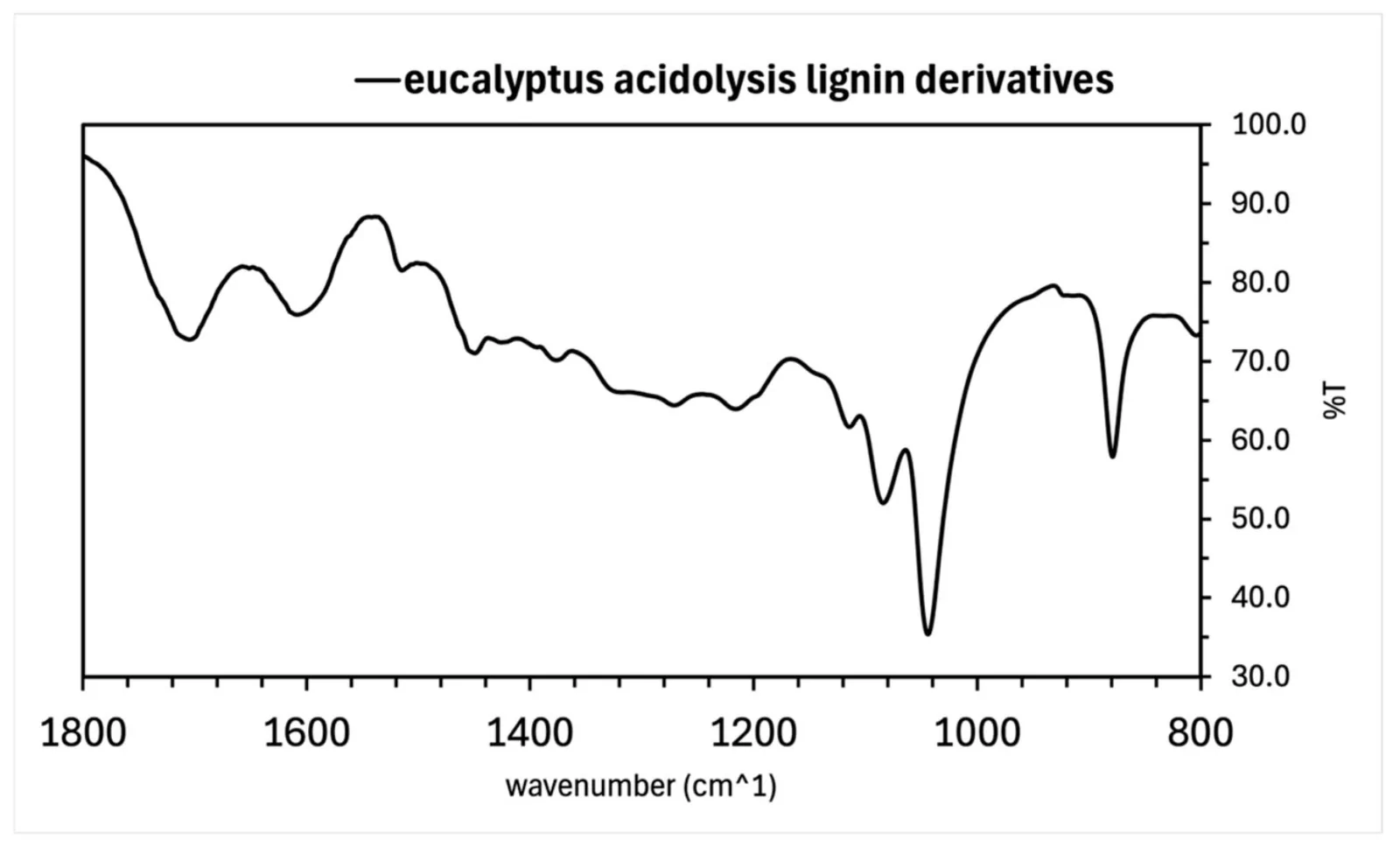
FTIR of the lignin derivatives fraction obtained after the mild acidolysis treatment from eucalyptus wood.

**Figure 3:**
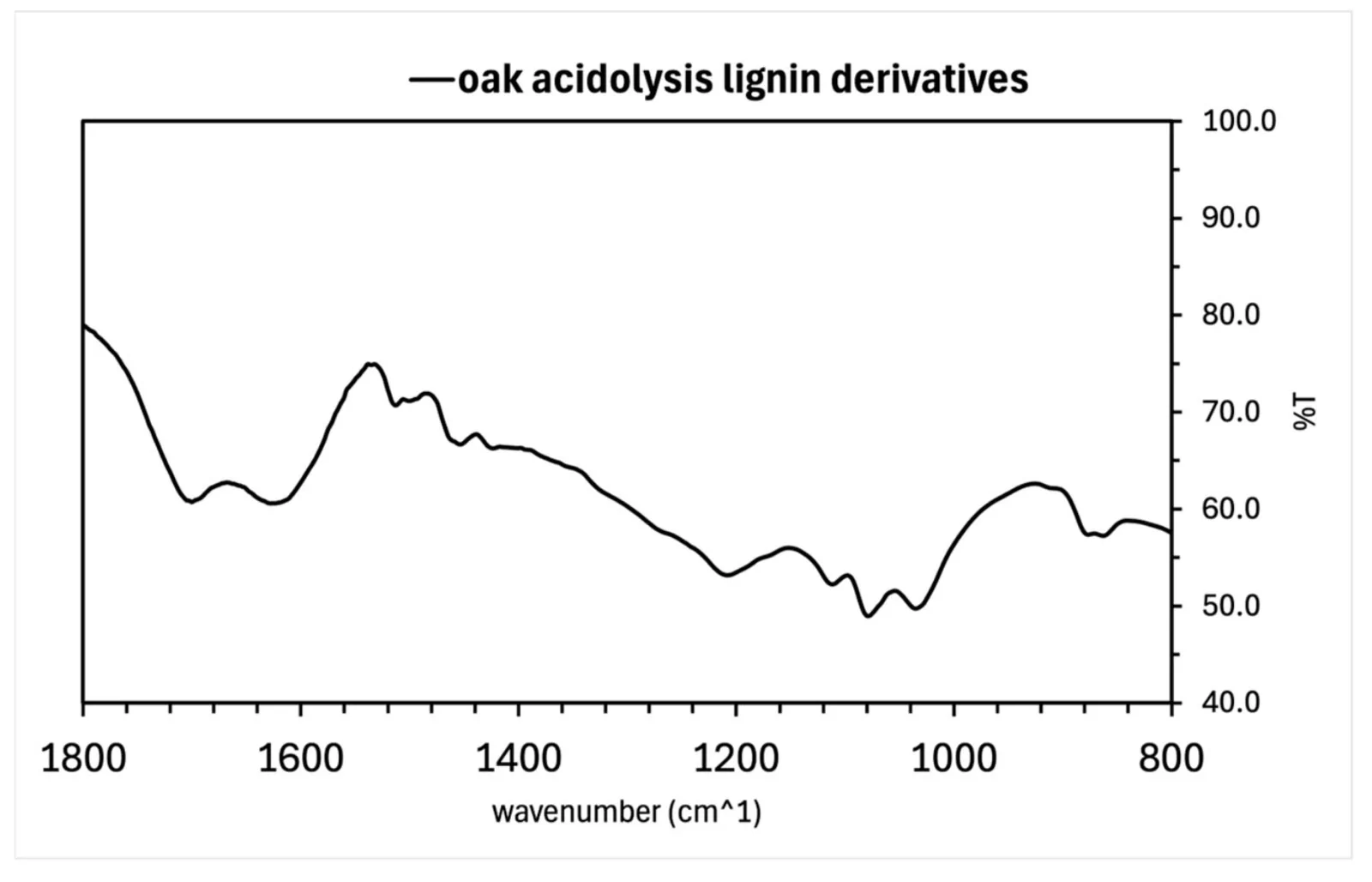
FTIR of the lignin derivatives fraction obtained after the mild acidolysis treatment from oak wood.

The FTIR spectra of the lignin-derivative fractions obtained from beech (BE), eucalyptus (EU), and oak (OA) hydrochars are shown in Figure 1, Figure 2 and Figure 3.

They all display, as summarized in Table 1, a set of bands that are consistent with the presence of lignin-related functional groups. All three samples show signals in the region associated with conjugated carbonyl structures, with bands between 1651 and 1666 cm^−1^ falling within the reference range for C=O stretching in conjugated p-substituted aryl ketones [39]. Additional carbonyl contributions are observed at 1627 cm^−1^ in BE and OA, which are compatible with lignin-derived structures [40]. The aromatic domain is evident across all samples: bands around 1531–1536 cm^−1^ correspond to aromatic skeletal vibrations [39,41], while those at approximately 1502–1509 cm^−1^ correspond to ring vibrations of lignin [42]. Signals in the 1478–1485 cm^−1^ region, assigned to C-H deformations of methyl and methylene groups, are present in all three spectra and indicate contributions from both lignin and residual carbohydrate structures [39,41]. Additional aromatic C-H vibrations are observed near 1440 cm^−1^, with all samples showing peaks close to the reference value of 1429 cm^−1^. Several bands associated with phenolic and guaiacyl-type structures are also detected: C-O stretching of phenolic groups appears around 1234–1240 cm^−1^, while the guaiacyl-related C-O vibration at 1205–1217 cm^−1^ is consistently present [40,42].

The region between 1129 and 1145 cm^−1^, typically attributed to in-plane aromatic C-H deformation of G-units, is represented in all three lignin derivatives, although with slight shifts among the species [39]. Finally, bands at 918–927 cm^−1^, corresponding to out-of-plane aromatic C-H deformation, further confirm the presence of aromatic moieties [39]. Taken together, the spectra of BE, EU and OA lignin derivatives share a common set of lignin-typical vibrations, with only minor shifts in peak positions among the three biomasses. A substantial proportion of the detected bands corresponds to aromatic and phenolic vibrations that are characteristic of lignin, confirming that the isolated fraction is enriched in lignin-derived structures. These findings indicate that the acidolysis treatment successfully released an aromatic component enriched in lignin-associated functional groups.

### 3.2. Solubility Tests

To assess the possible presence of residual polysaccharides in the EU, OA, and BE lignin derivatives sample, solubility tests were also performed using dimethyl sulfoxide (DMSO). The solubility of samples in DMSO suggests a limited content of insoluble polysaccharide residues; however, solubility alone does not allow their complete absence to be confirmed [43]. The samples were visually inspected after 24 h and were all found to be completely soluble.

### 3.3. High Performance Size Exclusion Chromatography (HP-SEC)

**Figure 4:**
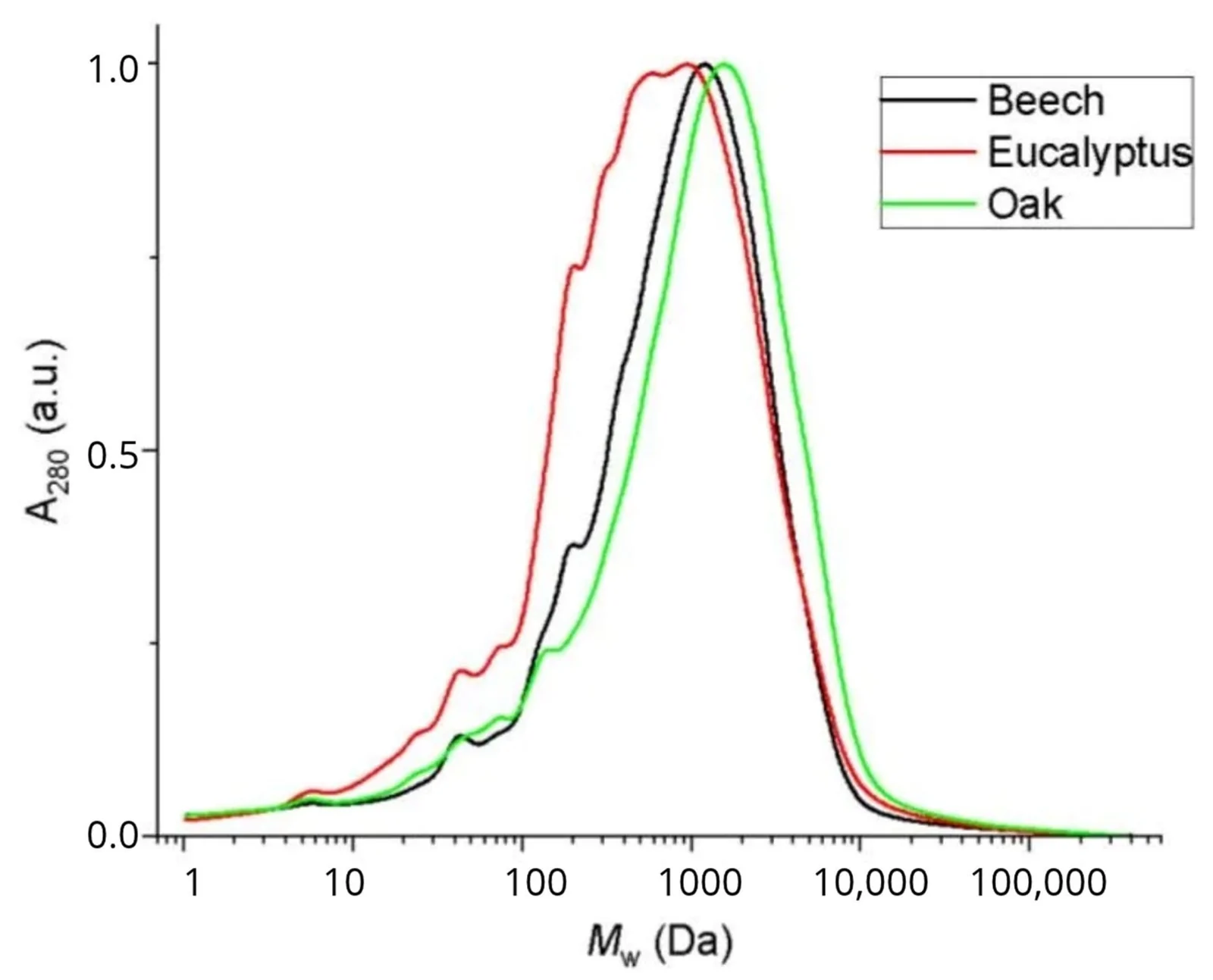
Molecular weight distributions (MWD) of the beech lignin derivatives (black), eucalyptus lignin derivatives (red), and oak lignin derivatives (green) obtained via mild acidolysis.

Although recognizing structural features consistent with lignin is an important first step, this information alone is not sufficient for its unambiguous identification. An additional key aspect is the molecular weight distribution of the lignin. For instance, lignin with relatively low molecular weight (typically 0.500–3 kDa) [17] can undergo oxidation to yield compounds such as vanillin or vanillic acid-valuable molecules widely used as flavoring agents–or can serve as a precursor for BTX aromatics, which today are predominantly produced from petroleum and account for roughly 60% of the global aromatic chemicals market. Low-molecular-weight lignin can also be incorporated into polyurethane foams [44]. Conversely, when the goal is to obtain functional nanoparticles, a much higher molecular weight–on the order of several kilodaltons–is generally required [45]. Results of the analysis are shown in Table 2 and Figure 4.

HP-SEC analysis of the lignin derivatives obtained from beech (BE), oak (OA), and eucalyptus (EU) reveals moderate differences in their molecular weight shown in Figure 4. The reported molecular weight values are relative estimates derived from calibration with polystyrene standards and are therefore intended for comparative evaluation among samples rather than as absolute molecular weight determinations. As summarized in Table 2, oak lignin derivatives exhibit the highest number-average and weight-average molecular weights (M_w_ = 2200 Da, M_n_ = 880 Da), followed by beech (M_w_ = 1700 Da, M_n_ = 830 Da) and eucalyptus (M_w_ = 1600 Da, M_n_ = 700 Da). The dispersity values (Đ) further indicate that oak derivatives possess the broadest molecular weight distribution (Đ = 2.4), while beech shows the narrowest (Đ = 2.0) and eucalyptus lies in between (Đ = 2.2). These numerical trends are consistent with the molecular weight distribution in Figure 4, where the oak sample displays a profile shifted towards higher weight-average molecular weights. In contrast, the eucalyptus sample shows a distribution shifted toward lower weight-average molecular weights, with a comparatively broader peak. The beech sample occupies an intermediate position both in terms of elution profile and weight-average molecular weight parameters. Overall, the combined analysis of chromatograms and HP-SEC-derived molecular weight data demonstrates that, within this fraction of lignin derivatives obtained from hydrothermal carbonization of virgin beech, oak, and eucalyptus biomass, oak-derived lignin derivatives are characterized by higher weight-average molecular weights and greater dispersity, whereas eucalyptus-derived derivatives are comparatively lower in molecular weight, with beech-derived materials exhibiting intermediate behavior.

### 3.4. 2D HSQC NMR

**Figure 5:**
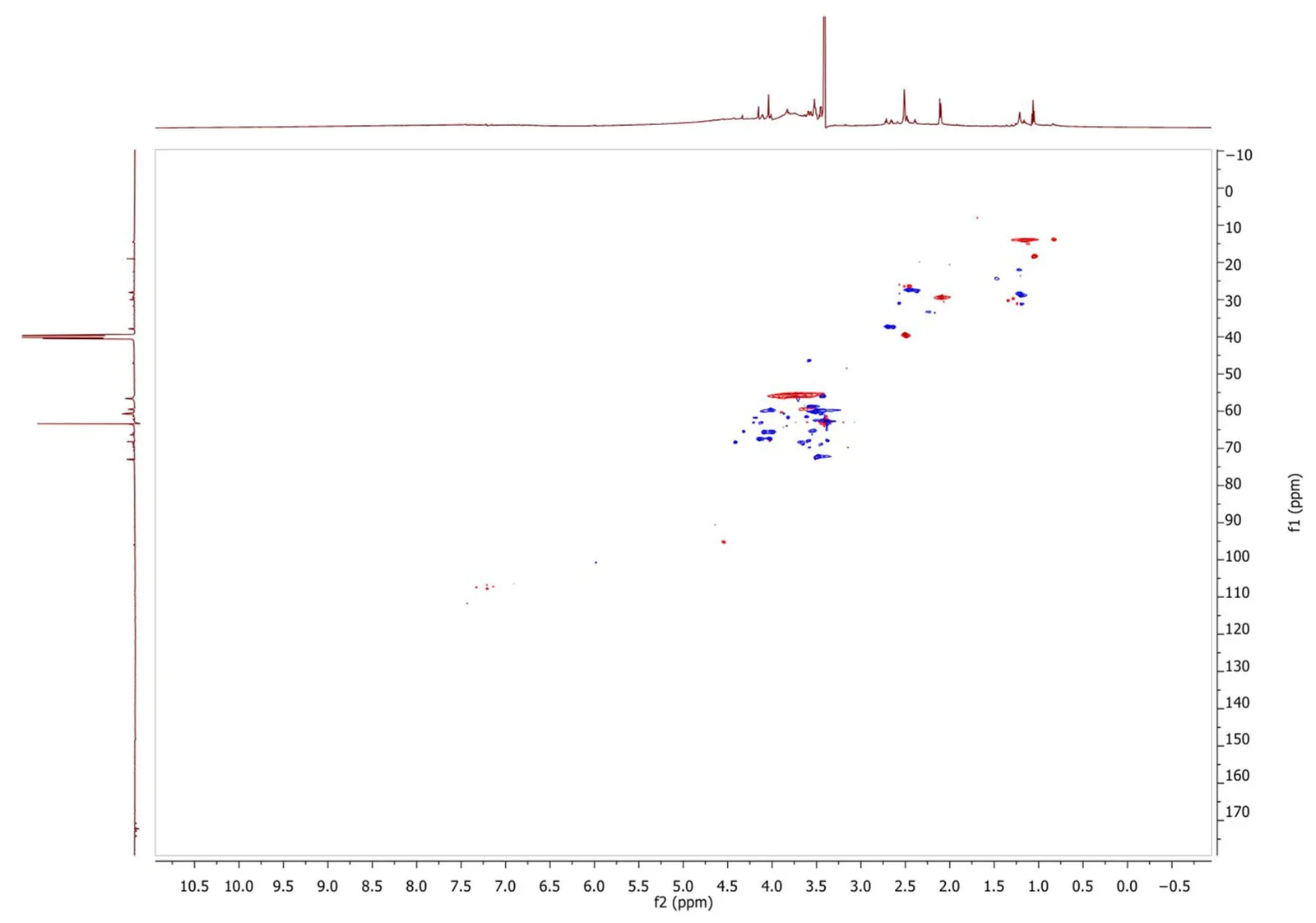
2D HSQC NMR of beech lignin derivatives. Red signals denote CH and CH_3_ resonances, whereas blue signals represent CH_2_ resonances.

**Figure 6:**
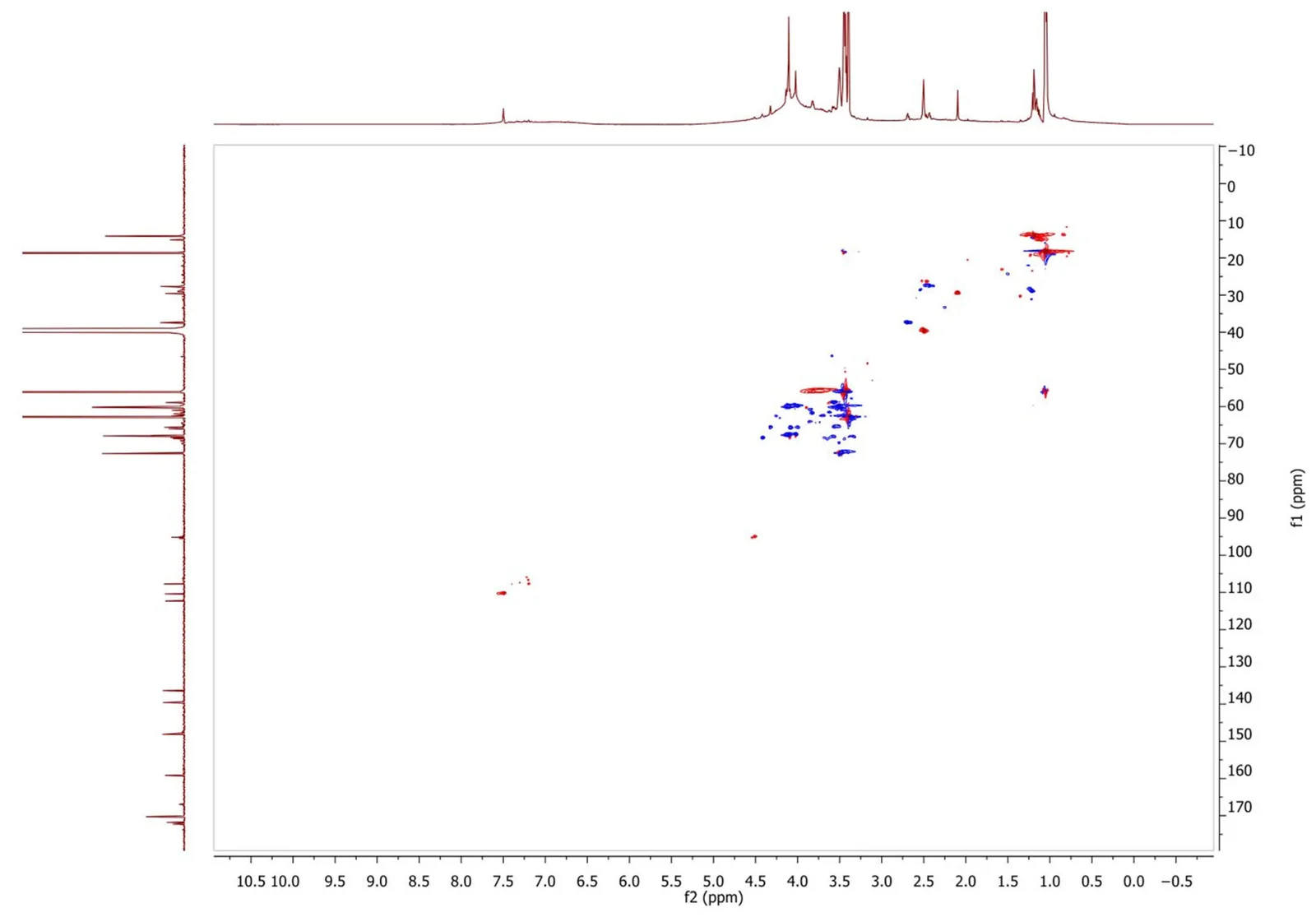
2D HSQC NMR of eucalyptus lignin derivatives. Red signals denote CH and CH_3_ resonances, whereas blue signals represent CH_2_ resonances.

**Figure 7:**
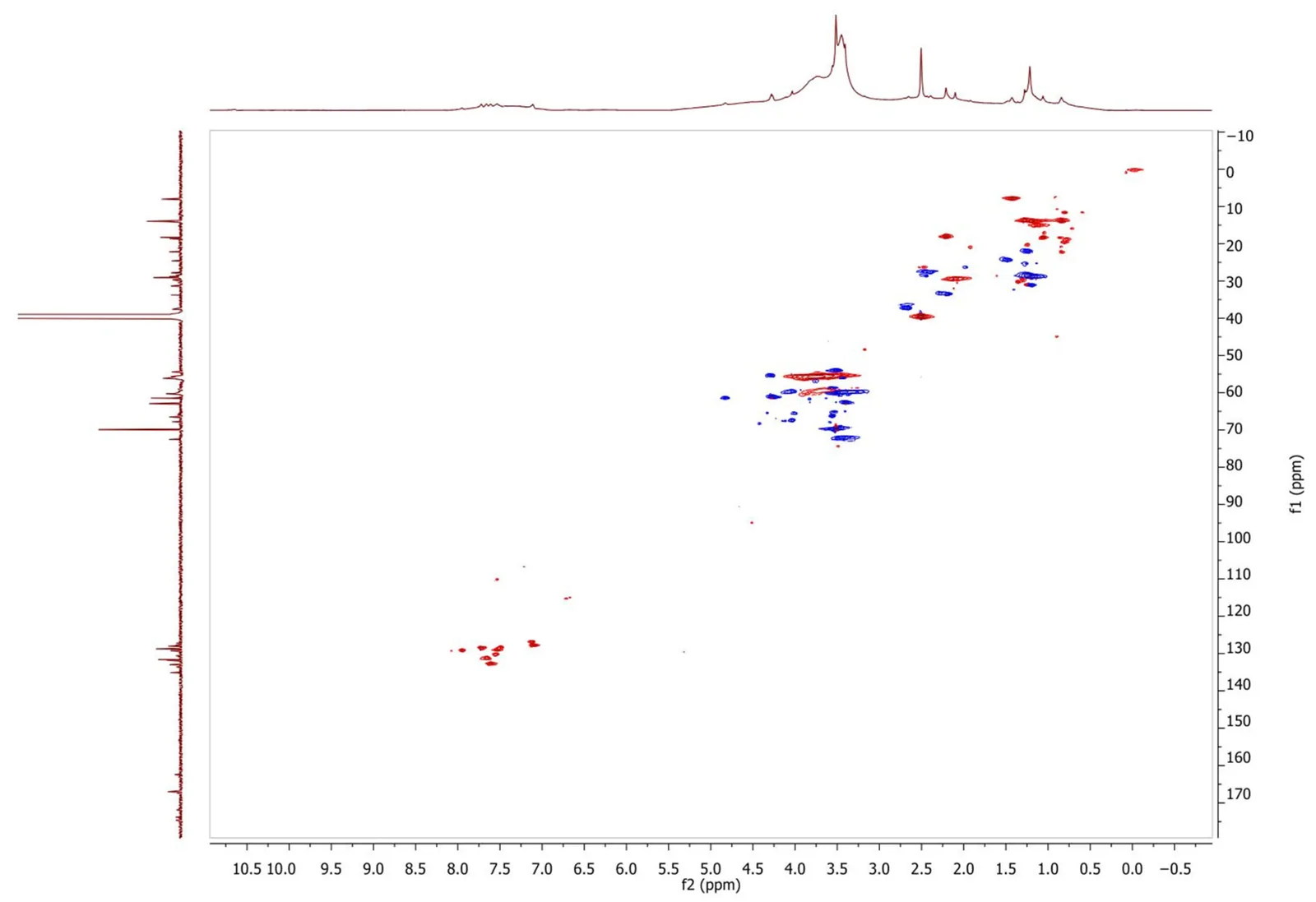
2D HSQC NMR of oak lignin derivatives. Red signals denote CH and CH_3_ resonances, whereas blue signals represent CH_2_ resonances.

2D HSQC NMR analysis of the samples was conducted, which confirms the presence of lignin and other derivatives, likely due to the extensive depolymerization/recondensation of phenolic materials in the original sample caused by the harsh conditions, as can be observed in Figure 5, Figure 6 and Figure 7.

For all the samples, the aromatic region exhibits correlation within 6.5–8.0 ppm (^1^H) and 100–120 ppm (^13^C), indicating aromatic units. These chemical-shift ranges correspond to aromatic carbons in guaiacyl (G) and syringyl (S) units in lignin. However, the oak sample displays a greater number of signals in this region. This higher spectral complexity can be primarily linked to the more structurally diverse and condensed native aromatic framework of oak, which preferentially retains cross-linked core structures during treatment, as further addressed in the comparative section “Lignin derivatives comparison” in the “2D HSQC NMR insights” subsection.

The region around 2.5–5.0 ppm (^1^H) and 50–90 ppm (^13^C) corresponds to oxygenated sp^3^ carbons, such as those found in alcohols, ethers, esters, or carbohydrate-like structures, where the attached protons are shifted downfield by the presence of a neighbouring oxygen atom, indicating that the sample contains a substantial proportion of oxygenated aliphatic functionalities. Here fall β-O-4 linkages resonances, containing several correlations that match the expected chemical-shift ranges for the different positions within this interunit motif: β-O-4γ appears between 3 and 4 ppm (^1^H) and between 60 and 65 ppm (^13^C) as well as at 4.5 ppm (^1^H) and 65 ppm (^13^C); β-O-4_β_ is represented by cross-peaks between 4 and 5 ppm (^1^H) and 80 and 85 ppm (^13^C);

β-O-4_α_ shows correlation cross-signals at around 5 ppm (^1^H) and 70 ppm (^13^C). The simultaneous presence of these three sets of resonances is a strong indicator of lignin in the sample, as the O-4 bond is the most abundant linkage in lignin. Although similar cross-signals may also arise from polysaccharide fragments, the spectra display a broad cross-signal between 3.5–4.0 ppm (^1^H) and 50–60 ppm (^13^C), consistent with methoxyl groups, which are characteristic of lignin and not of carbohydrates. Furthermore, the absence of significant signals in the 90–110 ppm (^13^C) aliphatic regions also supports the interpretation that the dominant contributions in this part of the spectra originate from lignin rather than carbohydrate residues.

A more localized signal around 90–100 ppm (^13^C) and 4.0–5.0 ppm (^1^H) for the eucalyptus sample is compatible with highly shifted oxygen-neighboring carbons, such as acetal or anomeric-type environments, as commonly found in carbohydrate-derived structures.

Notably, the spectra do not show cross-signals in the region between 4.0 and 7.0 ppm (^1^H) and 100 and 155 ppm (^13^C) would be expected, indicating the possible formation of condensed structures forming quaternary carbons that cannot be detected using HSQC analysis.

Nevertheless, the overall pattern of cross-signals demonstrated the aromatic lignin structure of the isolated fractions, confirming the lignin-derived nature of the material. The assignment of the cross-signals in the NMR spectra was carried out based on literature data [37,38,46,47,48,49,50,51].

### 3.5. Thermogravimetric Analysis (TGA)

**Figure 8:**
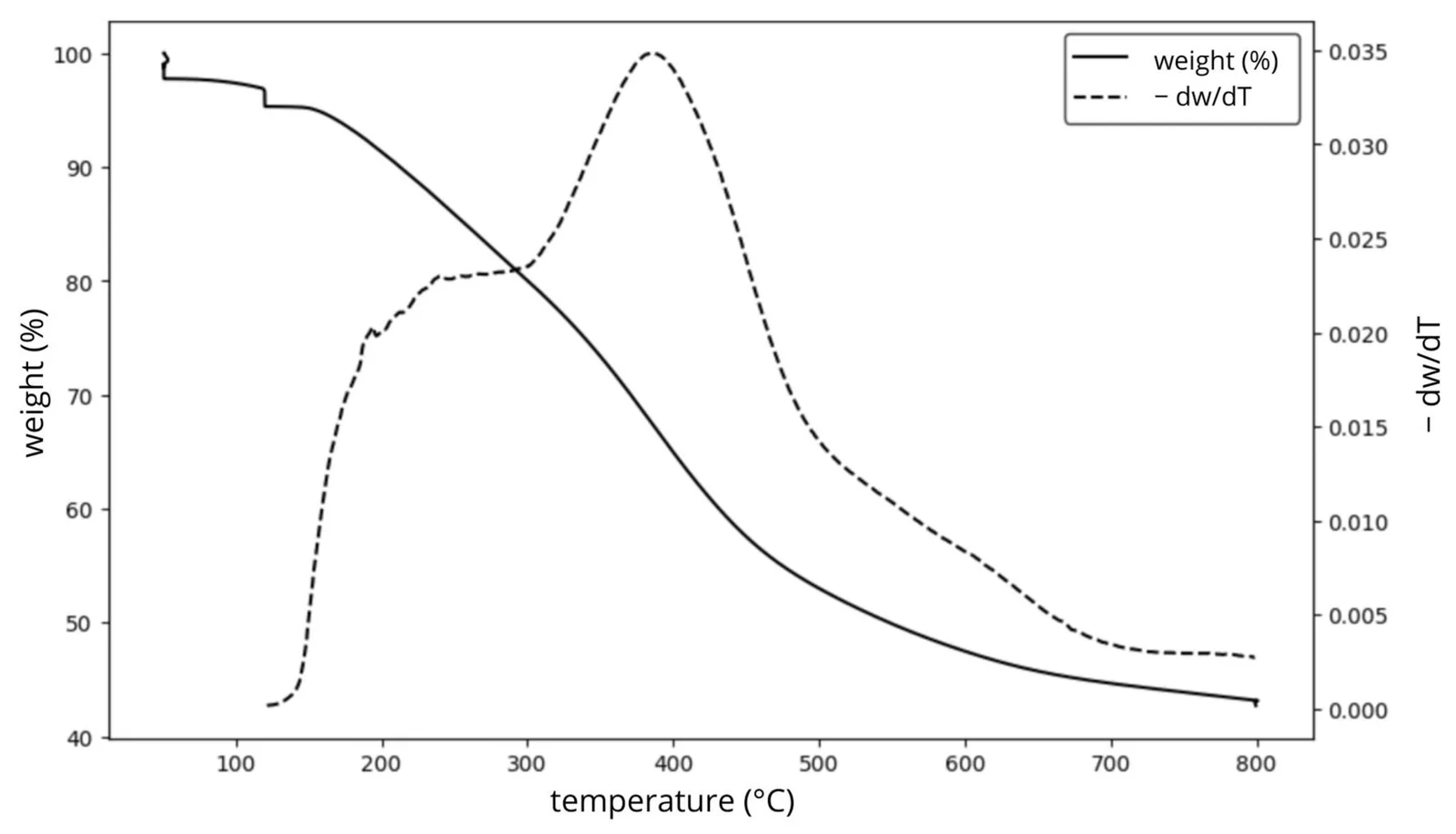
TGA of beech lignin derivatives.

**Figure 9:**
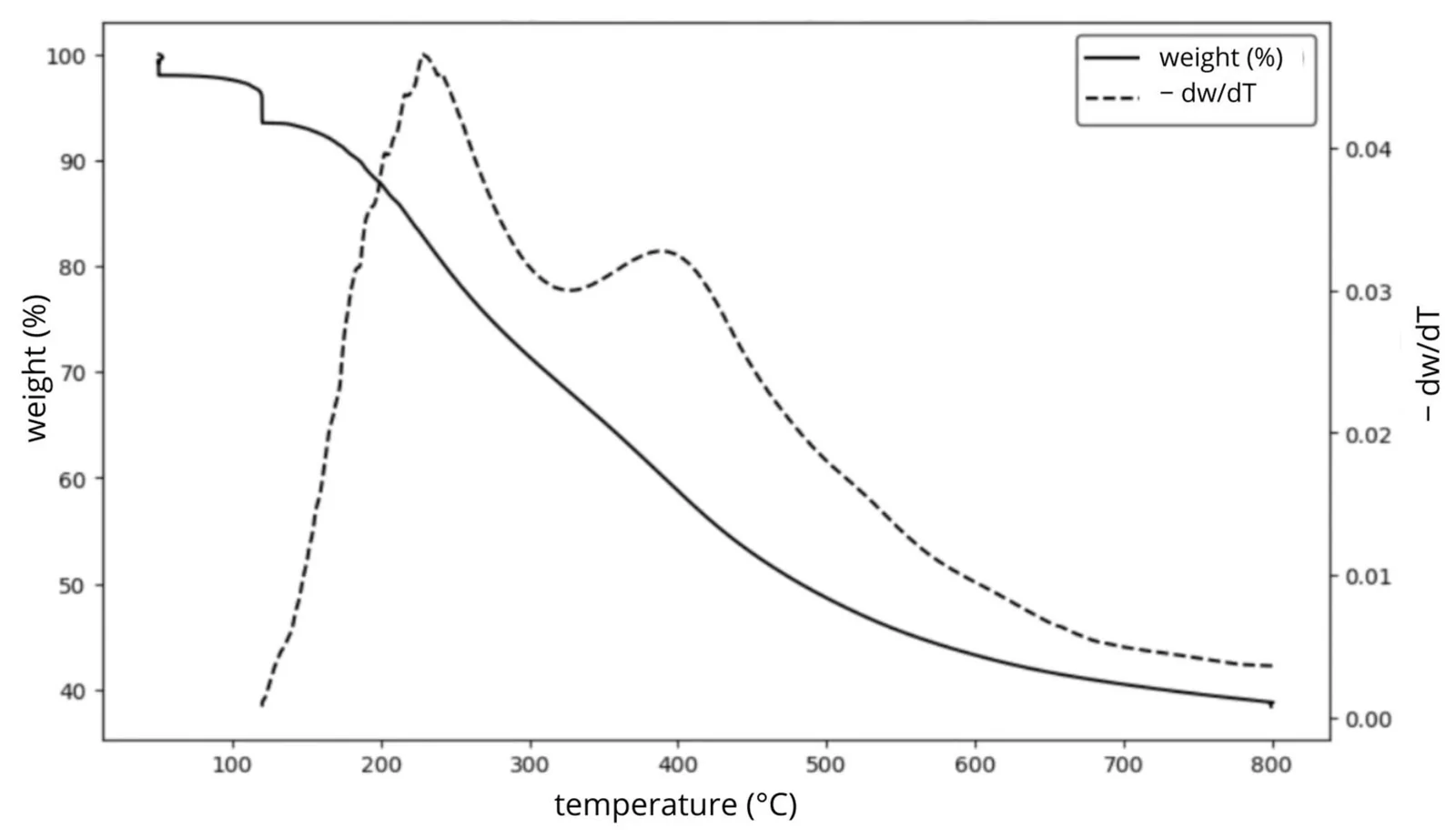
TGA of eucalyptus lignin derivatives.

**Figure 10:**
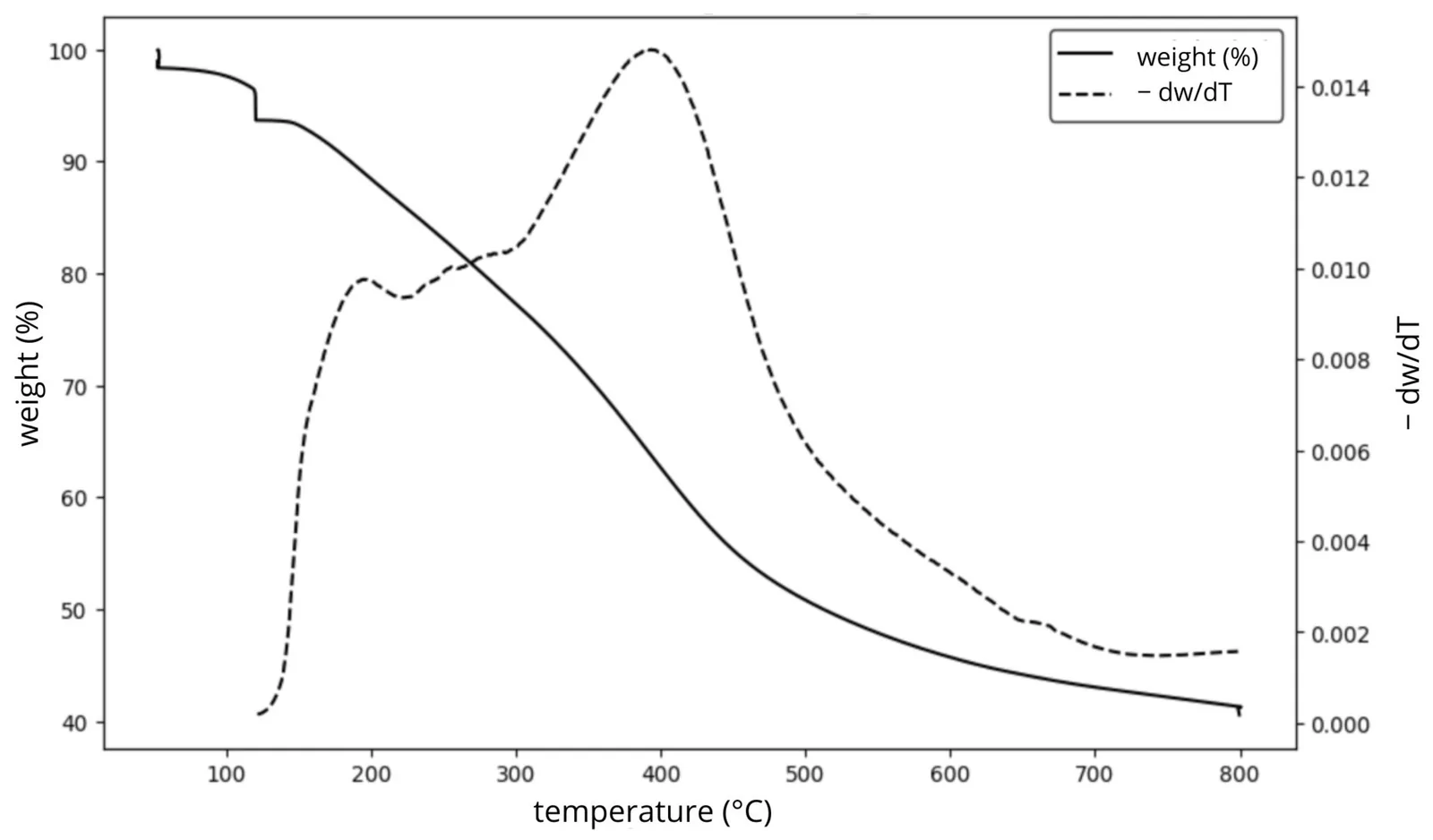
TGA of oak lignin derivatives.

The TGA graphs of beech, eucalyptus, and oak lignin derivatives from which the data were obtained are displayed in Figure 8, Figure 9 and Figure 10.

As shown in Table 3 and Table 4, TGA reveals a broadly similar thermal behavior among the three lignin derivatives from different biomasses. The temperature at 5% weight loss is highest for BE (155 °C), whereas EU and OA both start to decompose at lower temperatures (120 °C), indicating a slightly higher initial thermal stability for the beech-derived fraction.

Moreover, the %w at 800 °C in N_2_ provides insights into the decomposition stages and residues and reflects the proportion of the material that does not decompose under inert conditions. This value shows that all samples retain a substantial amount of mass, consistent with the formation of a carbonaceous residue in line with lignin-enriched materials. The % ashes at 800 °C in O_2_ represents inorganic or non-volatile components in the material, and it remains low (1–2%) for all samples.

T_peak_ represents the temperature at which the degradation reaches its maximum rate for each decomposition step, while T_onset_ stands for the temperature at which significant thermal degradation begins. The presence of two distinct values for the two main degradation events in nitrogen aligns with the NMR, HP-SEC, and FTIR results, which suggest the presence of a mixture of derivatives decomposing at different temperatures [11].

For each lignin-derivatives fraction, the onset of the first degradation step precedes the corresponding first derivative thermogravimetry peak, as expected, while the second peak occurs at significantly higher temperatures and is similar across samples, suggesting a common high-temperature degradation stage. Although distinct onset and peak temperatures can be identified, the TGA curves of all three lignin-derivative fractions do not exhibit any plateau region. Instead, the weight-loss profiles show a continuous slope throughout the heating program, indicating that mass loss occurs progressively rather than in sharply separated steps. This behavior also reflects the heterogeneous nature of the recovered fractions, in which components with different thermal stabilities degrade over partially overlapping temperature ranges.

Overall, the TGA data indicate that these lignin-derivative fractions show species-dependent initial thermal stability, with T_5%_ values ranging from 120 °C for EU and OA to 155 °C for BE, and undergo staged decomposition, leaving a significant carbonaceous residue at high temperature.

### 3.6. Lignin Derivatives Comparison

A comparative assessment of the lignin-derivative fractions isolated from beech (BE), eucalyptus (EU) and oak (OA). Moreover, the discussion was expanded to include lignin derivatives from chestnuts (CH) from the literature [11]. This work highlights how the botanical origin and the specific tissue chemistry of the starting hardwood govern their behavior during hydrothermal carbonization and subsequent mild solvolysis. The observed differences among species can be tentatively interpreted considering known variations in hardwood lignin structure, including differences in syringyl/guaiacyl ratio, degree of condensation, and cell-wall organization. Although these parameters were not directly quantified in the present work, literature data provide a useful framework for discussing the species-dependent trends observed in FTIR, HP-SEC, HSQC NMR and TGA analyses.

#### 3.6.1. Fingerprints FTIR Analysis

The infrared profiles demonstrated that all four biomasses yield solid fractions clearly enriched in aromatic frameworks, preserving the core skeleton of the native biopolymer (dominant bands around 1510–1515 cm^−1^ and 1600 cm^−1^ associated with aromatic skeletal vibrations and conjugated C=O stretching), which agrees with traditional isolated hardwood acidolysis lignins [1,52]. However, a key distinction emerges regarding the presence of non-lignocellulosic extractives. In the case of chestnut wood, the massive natural abundance of hydrolysable tannins acts as a significant interference during thermochemical processes [53]. The presence of these polyphenols in the non-detannized chestnut samples masks the typical lignin vibrations, requiring an industrial detannization step to properly resolve and emphasize the underlying lignin bands, such as the guaiacyl and syringyl breathing modes between 1320 and 1220 cm^−1^ [11]. Conversely, for BE, EU and OA, the characteristic hardwood lignin bands are directly prominent post-solvolysis without prior extractions. This implies that the structural accessibility or isolation purity of lignin in these three standard hardwoods is less constrained by heavily bound tannin networks.

#### 3.6.2. Molecular Weight Distributions

The macromolecular parameters determined by HP-SEC reveal a clear hierarchy in weight-average molecular weights: OA (2200 Da) > BE (1700 Da) > EU (1600 Da). In comparison, the chestnut lignin derivatives displayed a narrower but widely varying distribution depending on the presence of tannins [11]. The higher apparent M_w_ observed for OA may be directly correlated with its anatomical and chemical features as a dense, ring-porous hardwood. Oak is known to possess a highly branched and condensed native lignin network, characterized by a higher proportion of carbon-carbon interunit linkages, such as β-5′ and 5-5′ structures [52]. These condensed linkages are significantly more resistant to hydronium-catalyzed cleavage during HTC than the labile β-O-4′ ether bonds [54]. Therefore, under identical mild solvolysis conditions, the oak lignin may retain larger and cross-linked core lignin fragments, which could contribute to the higher apparent molecular weight observed in this study [52].

In contrast, EU, as a fast-growing species, is typically characterized by a high syringyl/guaiacyl ratio [55,56]. Because the C5 position on the aromatic ring of syringyl units is methoxylated, it is sterically hindered and cannot easily participate in C-C cross-linking. This linear, ether-bound architecture may render eucalyptus lignin more susceptible to cleavage during acid hydrothermal treatment [57], potentially contributing to the lower apparent molecular weights observed.

This behaviour aligns with literature reporting that eucalyptus wood fibres exhibit a high exposure of easily cleavable surface lignin structures depending on genotype and site conditions [58], and that its xylem/sapwood lignin is structurally less condensed than that found in more complex tissue parts like the bark [59].

#### 3.6.3. 2D HSQC NMR Insights

HSQC spectra confirm the preservation of β-O-4 linkages across all samples, accompanied by intense methoxyl cross-signals (δ_H_–δ_C_ 3.7–56 ppm), matching the typical fingerprint of hardwood lignins [1,52]. A critical correlation with the Folin-Ciocalteu assays [11] validated that the free phenolic hydroxyl content is close to zero in the chestnut (CH) fractions. This absence indicates that the severe acidic environment promotes rapid, concomitant competitive pathways: while the ether bonds are cleaved to generate transient free phenolic groups, these reactive intermediates immediately undergo solid-state recondensation and carbonization [11]. In EU, the presence of distinct cross-signals in the oxygenated aliphatic region suggested a higher retention of carbohydrate-derived pseudo-lignin or tightly bound xylan remnants, validating its distinct cell-wall biochemical footprint [59] compared to the more defined aromatic domains of BE and OA. However, these assignments remain tentative because the carbohydrate content was not directly quantified.

#### 3.6.4. Thermal Stability

Thermogravimetric parameters under an inert atmosphere show a divergent trend in initial thermal stability (T_5%_) and final char residue: BE (155 °C, 65% residue) > OA (120 °C, 63% residue) = EU (120 °C, 59% residue). For comparison, the virgin chestnut fractions exhibited an early thermal onset (T_5%_ = 97 °C) due to tannin decomposition, which shifted to 121 °C only after detannization [11].

The higher T_5%_ of BE at 155 °C may reflect a greater structural homogeneity and/or a lower content of thermally labile components in the isolated fraction [1]. However, these properties were not directly measured in the present study and should therefore be regarded as possible explanations for the observed behavior. The role of thermal-triggering impurities–such as low-temperature decomposing hemicellulose degradation products or highly volatile, unconjugated light extractives–prevents early weight loss. This interpretation is consistent with the lower dispersity (Đ = 2.0) of BE, which may indicate a more uniform and chemically stable fragment distribution. This high architectural integrity is consistent with the dense and regular cell-wall packing of beech wood [34], which favors an even distribution of lignin-carbohydrate complexes. Conversely, the earlier weight loss (120 °C) observed for EU and OA may be associated with the presence of more volatile fragments or thermally labile oxidized side chains generated during the HTC step, which act as triggers for early pyrolytic volatilization. Furthermore, while chestnut derivatives display a complex multi-stage profile due to the heavy heterogeneity of the tannin-lignin-char mix [11], BE, EU and OA exhibit a cleaner, more uniform staged pyrolytic volatilization.

### 3.7. Implications for Potential Applications

Taken together, the molecular weight distribution, structural features and thermal behaviour of the recovered fractions indicate which applications are more plausible, although these considerations remain tentative given the exploratory nature of the data. With M_W_ between 1600 and 2200 Da and Đ between 2.0 and 2.4, the fractions fall in the low-molecular-weight range associated with monomer-oriented valorisation and polyurethane foams [17,44], but below the size generally required to obtain nanoparticles by nanoprecipitation [45]. Such fractions could nevertheless be converted into nanoparticles after chemical modification, as shown for kraft lignin fractions of comparable molecular weight that self-assembled into nanoparticles after esterification with cholesterol [25]. As regards bio-based resins and adhesives, HSQC indicated preserved β-O-4 linkages and methoxyl groups, but also acid-induced recondensation, and the free phenolic hydroxyl content of the chestnut fractions obtained under the same conditions was close to zero [11]. Since condensed and poorly reactive lignin generally requires modification, such as phenolation or demethylation, to be used in adhesive [28,32], these fractions are more likely to act as precursors of modified resins than as direct phenol substitutes. Among the species, eucalyptus, with the lowest molecular weight, appears the most suitable for depolymerisation or modification routes; oak, with the highest molecular weight, is closest to the size range needed for nanoparticles; and beech, with the highest initial thermal stability, occupies an intermediate position.

From a thermal point of view, the low T_5%_ (120–155 °C) would limit the melt processing of these fractions in polymer composites or thermoplastic bio-based packaging unless their melt behaviour is improved by chemical modification [25]. Conversely, the high carbonaceous residue at 800 °C (59–65%) and the low ash content are consistent with their use as precursors of lignin-derived carbons, whose porosity can be engineered by activation or templating for energy-storage electrodes [29]. Packaging applications exploiting the antioxidant and antimicrobial properties of lignin [60,61,62,63] would require the phenolic content and colour of the fractions to be assessed, which was beyond the scope of this study. Overall, these fractions appear better suited as precursors for modified resins, nanoparticles after modification and carbon materials than for direct use in melt-processed composites.

## 4. Conclusions

The combined spectroscopic, chromatographic and thermal analyses demonstrate that the mild acidolysis applied to the hydrochars of beech, eucalyptus and oak effectively isolated fractions enriched in lignin-derived structures.

FTIR reveals a consistent set of aromatic and phenolic vibrations across all samples, confirming the presence of lignin-typical functional groups and indicating that the recovered materials retain the characteristic chemical features of lignin despite the harsh hydrothermal conditions. HP-SEC analysis further shows that the three biomasses yield lignin-derivative fractions with distinct molecular weight distributions, with oak lignin derivatives producing the highest average molecular weights and the broadest dispersity, beech lignin derivatives exhibiting intermediate values, and eucalyptus lignin derivatives yielding the lowest molecular weights. These differences reflect the intrinsic variability of the original feedstocks and their response to hydrothermal carbonization. 2D HSQC NMR provides additional structural insights, revealing correlations associated with β-O-4 linkages, methoxyl groups, and aromatic C-H units, thereby confirming the lignin origin of the isolated fractions. The spectra also highlight minor contributions from oxygenated aliphatic moieties and, in specific cases, carbohydrate-like environments, while reflecting the occurrence of acid-induced recondensation pathways through the formation of HSQC-invisible condensed structures. TGA shows that all samples exhibit comparable thermal behavior, with initial degradation occurring between 120 and 155 °C and substantial carbonaceous residues remaining at 800 °C, in line with lignin-rich compositions. The absence of plateau regions in the TGA curves further indicates that the fractions undergo continuous, multi-stage decomposition, reflecting their heterogeneous nature.

When contextualized with the comparative analysis, these results highlight how the intrinsic lignin architecture of each species governs its behaviour under HTC and subsequent mild acidolysis. Oak, which is generally reported to possess a more condensed and cross-linked lignin network, may yield higher-molecular-weight fragments and a richer aromatic fingerprint, whereas eucalyptus, with its syringyl-rich and less condensed lignin, undergoes more extensive cleavage and retains a greater proportion of oxygenated aliphatic or carbohydrate-derived domains. Beech, consistently, occupies an intermediate position. These species-dependent trends are consistent with the hypothesis that the HTC-acidolysis sequence amplifies the native chemical signatures of each biomass, shaping both the molecular weight distribution and the degree of structural preservation.

The comparison with chestnut further reinforces the role of extractives and matrix composition in determining solvolysis efficiency, with previous chemical assays revealing a near-zero free phenolic content that aligns with the highly condensed nature of these hydrochar-derived fractions. While chestnut required detannization to reveal lignin-specific vibrations, the three species examined here displayed a clear lignin fingerprint without prior extraction, indicating a more accessible lignin domain.

Overall, the results demonstrate that hydrothermal carbonization followed by mild acidolysis yields lignin-enriched fractions whose structural, molecular, and thermal properties depend on the botanical origin of the biomass. The present work was primarily intended as a qualitative characterization of the recovered lignin derivative fractions. Therefore, quantitative process metrics such as hydrochar yield, lignin recovery efficiency, and overall mass balances were beyond the scope of the study and should be considered in future investigations focused on process evaluation and scale-up.

These findings highlight both the potential and the limitations of the current approach. While the method successfully extracts an aromatic, lignin-rich fraction, the degree of structural heterogeneity suggests that there is significant room for improvement in developing milder or more selective strategies, so as to recover lignin with higher structural integrity and purity–essential for its use in higher-value-added applications such as advanced materials. On the basis of their relatively low molecular weight and residual condensation, the recovered fractions appear more suitable as precursors for modified bio-based resins, nanoparticles after modification, and carbon materials than for direct use in melt-processed composites. In this sense, the present work provides a solid foundation while clearly pointing toward future developments needed to fully unlock the potential of lignin recovered from hydrothermal carbonization processes.

## Figures and Tables

**Table 1:** Main functional groups and peaks of beech (BE), eucalyptus (EU) and oak (OA) lignin derivatives after the mild acidolysis treatment.

Wavenumber (cm^−1^)				Base Group and Vibration Mode
BE	EU	OA	reference	
1660	1651	1666	1675–1655	C=O stretch in conjugated p-subst. aryl ketones [39]
1627			1627	C-O ketone [40]
1534	1536	1531	1515–1505	aromatic skeletal vibrations plus C=O stretching [39,41]
1503		1509	1502	aromatic skeletal vibrations [42]
1489	1485	1478	1470–1460	C-H deformations stretching in lignin and carbohydrates, C-H deformation (methyl and methylene) [39,41]
1453		1461	1463	CH_2_ deformation stretching in lignin and xylan [41]
1440	1431	1441	1429	C-H vibration for aromatic ring [40]
1366	1385		1375–1372	aliphatic C-H stretching in methyl and phenol OH or O-H vibration for phenolic groups [39]
1260			1270–1266	G ring plus C=O stretch [39]
1234	1240		1236	C-O stretching of phenol [40]
1210	1205	1217	1218	C-O of guaiacyl ring [42]
1177	1163		1166	typical for HGS lignins; C=O in ester groups, conj. [39]
1158			1160	C-O-C stretching in pyranose rings, C=O stretching in aliphatic groups [41]
1129	1135	1145	1140	aromatic C-H in plane deformation, typical for G units; plus C=O stretch [39]
1095	1102	1096	1102	O-C=O stretching [40]
1075	1060	1061	1052	O-H stretching for primary alcohol and C-O-C stretching [40]
918	927	927	925–915	C-H out of plane; aromatic [39]
899	898	897	896	β-glucosidic bonds between sugars, character of cellulose P-chains, C-H stretching out of plane of aromatic ring [40,41]

**Table 2:** Results obtained via HP-SEC concerning the molecular weight.

Sample	M_w_ (Da)	M_n_ (Da)	Đ
BE lignin derivatives	1700	830	2.0
OA lignin derivatives	2200	880	2.4
EU lignin derivatives	1600	700	2.2

**Table 3:** Results concerning the TGA of the lignin derivatives sample of BE, EU and OA. T_5%_ is the temperature (°C) at which 5% of the material’s initial weight has been lost. %w 800 °C _N2_ indicates the percentage of the material’s weight remaining at 800 °C; %ashes 800 °C _O2_ indicates the residue left after complete thermal decomposition.

Sample	T_5%_	%w 800 °C _N2_	%ashes 800 °C _O2_
BE	155	65	2
EU	120	59	1
OA	120	63	1

**Table 4:** Results concerning the TGA of the lignin derivatives sample of BE, EU and OA. All the T values are in °C. T_N2__onset_ and T_N2 peak_ denote the onset temperature and the temperature of maximum mass-loss rate (DTG peak) measured under N_2_ atmosphere, respectively. Labels 1 and 2 refer to the first and second thermal degradation steps.

	BE	EU	OA
T_N2 peak;__1_	197	188	231
T_N2 onset; 1_	160	154	151
T_N2 peak; 2_	385	395	387

## Data Availability

The raw data supporting the conclusions of this article will be made available by the authors on request.
